# Automatic Diagnosis of Rice Diseases Using Deep Learning

**DOI:** 10.3389/fpls.2021.701038

**Published:** 2021-08-19

**Authors:** Ruoling Deng, Ming Tao, Hang Xing, Xiuli Yang, Chuang Liu, Kaifeng Liao, Long Qi

**Affiliations:** ^1^College of Engineering, South China Agricultural University, Guangzhou, China; ^2^Lingnan Guangdong Laboratory of Modern Agriculture, Guangzhou, China

**Keywords:** convolutional neural network, rice disease, ensemble learning, diagnosis, deep learning

## Abstract

Rice disease has serious negative effects on crop yield, and the correct diagnosis of rice diseases is the key to avoid these effects. However, the existing disease diagnosis methods for rice are neither accurate nor efficient, and special equipment is often required. In this study, an automatic diagnosis method was developed and implemented in a smartphone app. The method was developed using deep learning based on a large dataset that contained 33,026 images of six types of rice diseases: leaf blast, false smut, neck blast, sheath blight, bacterial stripe disease, and brown spot. The core of the method was the Ensemble Model in which submodels were integrated. Finally, the Ensemble Model was validated using a separate set of images. Results showed that the three best submodels were DenseNet-121, SE-ResNet-50, and ResNeSt-50, in terms of several attributes, such as, learning rate, precision, recall, and disease recognition accuracy. Therefore, these three submodels were selected and integrated in the Ensemble Model. The Ensemble Model minimized confusion among the different types of disease, reducing misdiagnosis of the disease. Using the Ensemble Model to diagnose six types of rice diseases, an overall accuracy of 91% was achieved, which is considered to be reasonably good, considering the appearance similarities in some types of rice disease. The smartphone app allowed the client to use the Ensemble Model on the web server through a network, which was convenient and efficient for the field diagnosis of rice leaf blast, false smut, neck blast, sheath blight, bacterial stripe disease, and brown spot.

## Introduction

Rice is an important crop in agriculture. However, crop diseases can significantly reduce its yield and quality, which is a great threat to food supplies around the world. Thus, disease control is critical for rice production. The key for successful disease control is a correct and fast diagnosis of diseases, so that pesticide control measures can be applied timely. Currently, the most widely used method to diagnose rice crop diseases is manual judgment based on the appearance of diseases (Sethy et al., 2020). There are not enough people across the region with skills to perform such tasks in a timely manner. Therefore, a more efficient and convenient method for disease diagnosis of rice is required.

Over the past decades, researchers have used computer vision technology in agriculture for estimating crop yields (Gong et al., 2013; Deng et al., 2020), detecting crop nutritional deficiencies (Xu et al., 2011; Baresel et al., 2017; Tao et al., 2020), estimating geometric sizes of crop (Liu et al., 2019), and recognizing weeds (Jiang et al., 2020). Several different approaches of computer vision have also been used for the diagnosis of crop diseases, such as image processing, pattern recognition, support vector machine, and hyperspectral detection (Ngugi et al., 2020). Multi-spectral remote sensing images of tomato fields were used for cluster analysis to differentiate healthy tomatoes from diseased ones (Zhang et al., 2005). The shape and texture features of rice bacterial leaf blight, sheath blight, and blast were extracted using a support vector machine. A genetic algorithm and a support vector machine were used to detect the diseased leaves of different crops (Singh and Misra, 2017). Islam et al. (2018) detected the RGB value of an affected portion, and then used Naive Bayes to classify rice brown spot, bacterial blight, and blast. Infrared thermal imaging technology that provides temperature information of crop has also been used to detect tomato mosaic disease and wheat leaf rust (Zhu et al., 2018). Although some of these existing methods could achieve reasonably high accuracies for crop disease diagnosis, most of them rely on manual extraction of disease features. As a result, the expression ability is limited, and it is difficult to generalize when results are applied. Also, some methods need special equipment that is not always readily available to users. All these drawbacks make it difficult to apply these methods for crop disease diagnosis.

Deep learning technology can be implemented in crop disease diagnosis methods to overcome the drawbacks. In recent years, deep learning has been widely used in image classification, object detection, and content recommendation. In fact, there have been researchers who used deep learning to detect diseases of various crops. Lu et al. (2017a) proposed an in-field automatic disease diagnosis system, which could achieve identification and localization for wheat diseases. Ozguven and Adem (2019) first applied a convolutional neural network (CNN), Faster R-CNN, to images of sugar beet leaves to detect spot disease. Karlekar and Seal (2020) proposed SoyNet that was applied to soybean leaf images for disease diagnosis. Deep learning also plays an important role in disease diagnosis of many other crops, such as tomato (Rangarajan et al., 2018; Agarwal et al., 2020), cassava (Sambasivam and Opiyo, 2020), tulip (Polder et al., 2019), and millet (Coulibaly et al., 2019). Deep learning has also been applied for detecting rice crop diseases. For example, Kamal et al. (2019) combined a depthwise separable convolution architecture with Reduced MobileNet. In terms of recognition accuracy, there have been various claims. Chen et al. (2020) used Enhanced VGGNet with Inception Module through migration learning, which had an accuracy of 92% in the classification of rice diseases. Rahman et al. (2020) proposed a two-stage small CNN architecture, which achieved 93.3% accuracy with smaller model sizes. Some efforts have been made to improve the accuracy. For instance, Picon et al. (2019) used a dataset of five crops, 17 diseases, and 121,955 images, then proposed three different CNN architectures that incorporate contextual non-image meta-data. Arnal Barbedo (2019) proposed a method of image classification based on individual lesions and spots, testing 14 plants and 79 diseases, which improved the accuracy compared with using original images.

Relying on a single predictive model may cause machine learning algorithm to overfit (Ali et al., 2014; Feng et al., 2020). To solve this problem, ensemble learning with a set of algorithms to combine all possible predictions was used (Dietterich, 2000). With the development of computer technology, ensemble learning was used for prediction in disease diagnosis (Albert, 2020), soybean yield (Yoosefzadeh-Najafabadi et al., 2021), protein binding hot spots (Hu et al., 2017), and wheat grain yield (Fei et al., 2021). Since the above studies have proven the feasibility of ensemble learning, ensemble technology would be used in this research to improve the accuracy of disease diagnosis.

In summary, deep learning is a promising technology for disease diagnosis of various crops with which high accuracy can be achieved. Existing research on the use of deep learning for rice diseases dealt with a limited number of rice diseases. Various types of rice diseases have been observed in rice fields, such as rice leaf blast, false smut, neck blast, sheath blight, bacterial stripe disease, and brown spot. The aim of this study was to increase the accuracy, efficiency, affordability, and convenience of rice disease diagnosis. The specific objectives of this study were to (1) develop a deep learning network model for diagnosing six different types of rice diseases, (2) evaluate the performance of the model, and (3) implement the diagnosis method in a cloud-based mobile app and test it in an application.

## Materials and Methods

### Model Development and Testing

#### Data Acquisition

Deep learning requires a large number of training images to achieve good results (Barbedo, 2018). Thus, a total of 33,026 images of rice diseases were collected over a 2-year period for the development of a disease diagnosis model. Among these images, 9,354 were for rice leaf blast, 4,876 were for rice false smut, 3,894 were for rice neck blast, 6,417 were for rice sheath blight, 6,727 were for rice bacterial stripe, and 1,758 were for rice brown spot diseases. The characteristics of rice leaf blast are large spindle-shaped lesions with grayish centers and brown edges. For false smut disease, the pathogen is fungal that infects rice flowers and turns them into rice false smut balls, which is the only visible feature of rice false smut. For rice neck blast disease, node and neck lesions often occur at the same time and have a similar characteristic, a blackish to a grayish brown color. For rice sheath blight disease, lesions on the leaves are usually irregular in shape, and after a period of infection, the center is usually grayish white with brown edges. For rice bacterial stripe disease, on young lesions, the bacteria ooze dew and dry out the plant, leaving yellow beads that eventually develop orange-yellow stripes on the leaves. For rice brown spot disease, the spots are initially small round, dark brown to purplish brown, and fully developed spots are round to elliptic with light brown to gray centers and reddish-brown edges. Example images of each disease are in the Supplementary Material. The images were from four locations in China: (1) Baiyun Base of The Guangdong Academy of Agricultural Sciences, Guangzhou, Guangdong, (2) Laibin, Guangxi, (3) Binyang, Guangxi, and (4) the Chinese Academy of Sciences, Hefei, Anhui. These images were taken using mobile phones with high resolution (more than 1 megapixel), so that the characteristics of rice diseases could be clearly captured. To prepare for model development, the images were split into a training set, a validation set, and a test set with a ratio of 7:2:1. This ratio was randomly applied to all the six disease categories; thus, the corresponding image numbers of these sets were 23,096; 6,684; and 3,246.

#### Image Preprocessing

**Figure 1:**
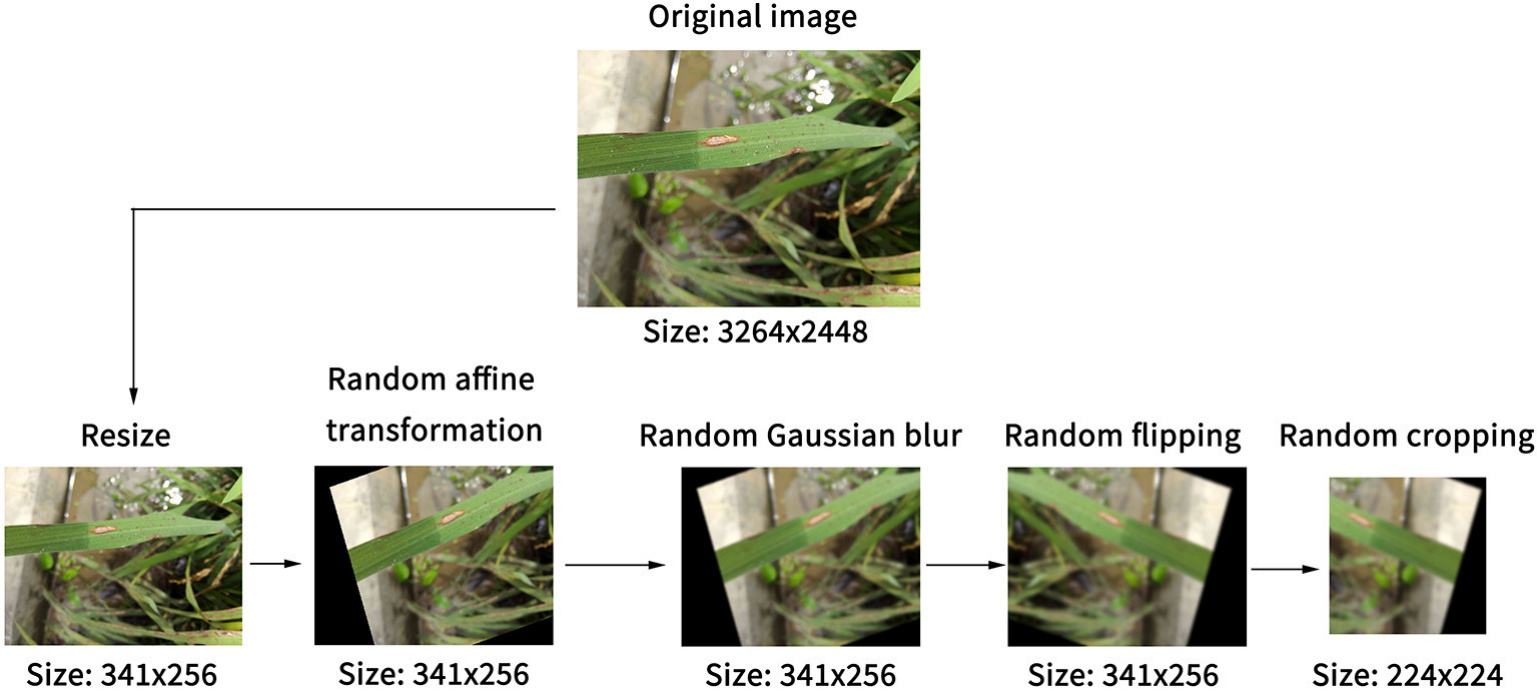
Steps of the image preprocessing for expanding dataset and reducing the overfitting of models.

Image preprocessing and data enhancement are performed to reduce the overfitting of models, as illustrated in Figure 1. Before the model reads the image, the short side of the image was scaled to 256 pixels, and the long side was scaled proportionally to reduce the computational pressure of the model. Then, random affine transformation was applied to the image, which could randomly translate, rotate, scale, deform, and cut the image. At the same time, Gaussian blur and image flipping were applied randomly. Finally, the resized image was randomly cropped to a 224 × 224 pixels square area as the actual training image. These processes favored expanding the data set and reducing the over-fitting of the model on the original dataset without modifying the characteristics of rice diseases.

Next, the mean and standard deviation of the ImageNet dataset were applied for normalization to make image color distribution as similar as possible. As the number of images of different types of diseases was not equal, an over-sampling operation was adopted for a small number of rice brown spot images in the preprocessing, with a ratio of three times. This process was repeated for each training epoch; therefore, the number of images that each model read was different in each training epoch, and the number of image samples in the dataset was increased in this way.

#### Convolutional Neural Network (CNN) Models

The structure of the convolutional neural network has a crucial influence on the performance of the final model. It was necessary to compare the performance of different networks in the diagnosis of rice diseases. Five network structures were selected and tested, and they were: ResNet, DenseNet, SENet, ResNeXt, and ResNeSt. These networks are described below.

**Figure 2:**
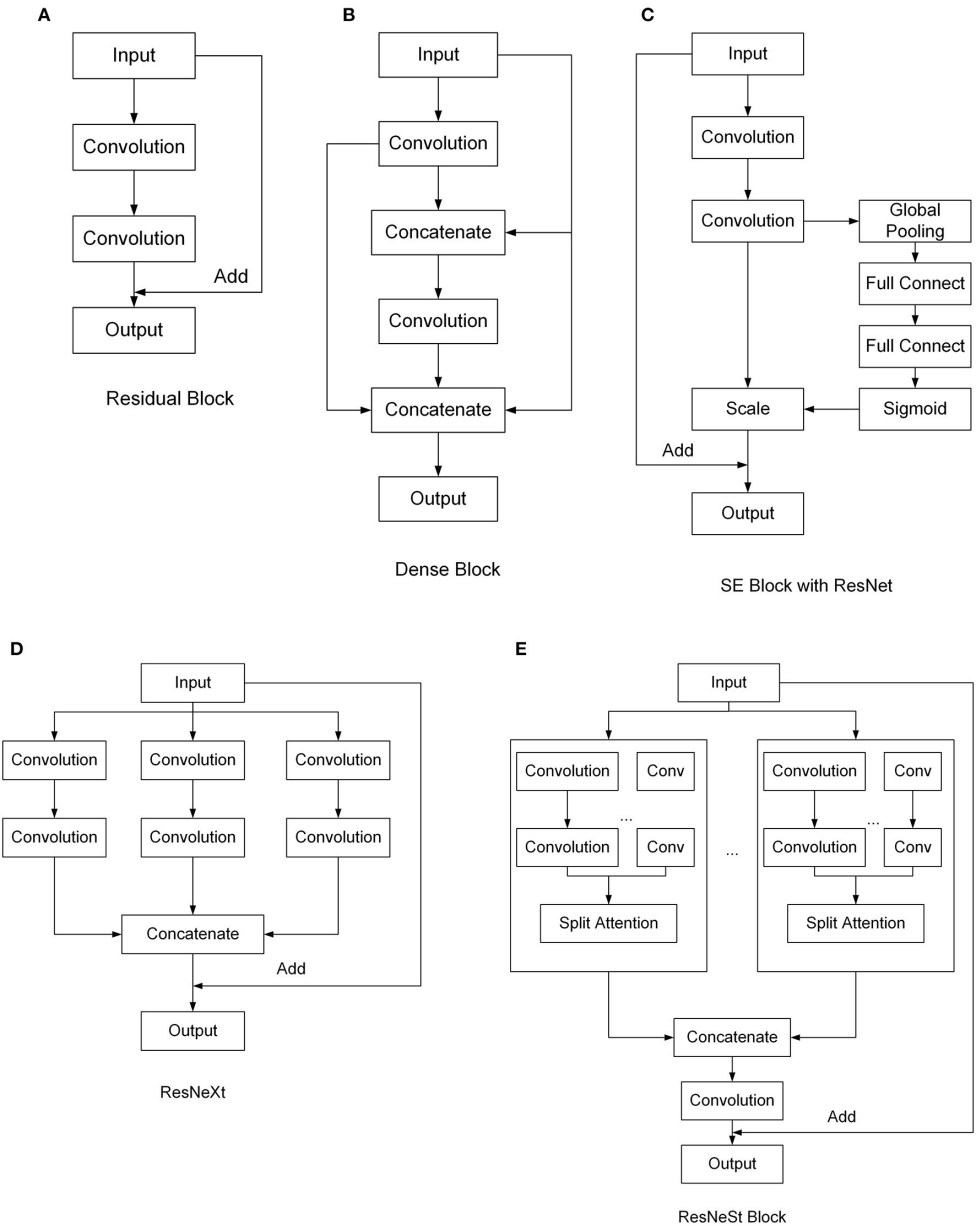
Structures of different convolutional neural network (CNN) models tested. **(A)** Residual Block, **(B)** Dense Block, **(C)** SE Block with ResNet, **(D)** ResNeXt, **(E)** ResNeSt Block.

ResNet (He et al., 2016) is a widely used network model, which uses residual blocks to enhance the depth of the CNN. The structure of the residual block is shown in Figure 2A. By directly connecting the input and the output, ResNet can reduce the problems of gradient disappearance and gradient explosion, thus deepening the number of network layers and achieving better effects. DenseNet (Huang et al., 2017) uses a dense connection, which connects each layer to every other layer (Figure 2B). Since DenseNet allows features to be reused, this can generate many features with a small number of convolution kernels. As a result, it can reduce gradient loss and enhance the propagation of features, and the number of parameters is greatly reduced. SE-ResNet (Hu et al., 2020) presents the “Squeeze-and-Excitation” block, which can establish the relationship between channels and adaptively recalibrate the responses of the channel-wise feature. The SE block can be added in different networks. Figure 2C shows the SE block with ResNet. ResNeXt (Xie et al., 2017) is an improved version of ResNet that was designed to have a multi-branch architecture and grouped convolutions to make channels wider (Figure 2D). ResNeXt can improve accuracy without increasing parameter complexity while reducing the number of super parameters. ResNeSt (Zhang et al., 2020) proposes Split-Attention blocks based on SENet, SKNet, and ResNeXt, which makes attentions grouped (Figure 2E). This structure combines channel attention and feature map attention to improve performance without increasing the number of arguments.

Based on the five network structures above, five network models were selected for subsequent training, and they were ResNet-50, DenseNet-121, SE-ResNet-50, ResNeXt-50, and ResNeSt-50. The MACs (multiply–accumulate operation number) and Params of the five network models above are shown in Table 1. MACs is an evaluation index of the computational force of the model, and Params is used to count the number of model parameters. Except for DenseNet-121, the number of calculations and parameters of the other models is very close. This means that their speed and model size are close to each other. Despite the small number of Params and MACs in DenseNet-121, due to the reuse of features, the occupation of training resources is still close to the other models, but it is more economical in model inference. Therefore, comparing these network models could eliminate the negative effect of hardware resource utilization.

**Table 1:** Parameters of the models.

**Model**	**MACs (G)**	**Params (M)**
ResNet-50	4.109	23.520
DenseNet-121	2.865	6.960
SE-ResNet-50	4.118	26.035
ResNeXt-50	4.257	22.992
ResNeSt-50	5.398	25.447

#### Evaluation of the Models

The performance of the five network models was compared, so that the best models could be selected. For each network model, the results of disease prediction were given in four categories, and they were true positive (TP): correctly predicted the type of disease; false positive (FP): other types of diseases were predicted as this disease; true negative (TN): correctly predicted the disease not being other types of disease; and false negative (FN): the disease was predicted to be another type of disease. These outputs were used to determine the performance indicators: accuracy, precision, recall rate, F1 score, and Matthews correlation coefficient (MCC), as shown in Equations (1–5). The accuracy and MCC were evaluated for all the types of diseases, and the other indicators were evaluated for a single type of disease:

(1) $$\begin{array}{r} A=\frac{\overset{6}{\underset{i=1}{\sum }}TP_{i}}{N} \end{array}$$

(2) $$\begin{array}{r} P_{i}=\frac{TP_{i}}{TP_{i}+FP_{i}} \end{array}$$

(3) $$\begin{array}{r} R_{i}=\frac{TP_{i}}{TP_{i}+FN_{i}} \end{array}$$

(4) $$\begin{array}{r} F1_{i}=\frac{2\cdot P_{i}\cdot R_{i}}{P_{i}+R_{i}} \end{array}$$

(5) $$\begin{array}{r} MCC=\frac{TP*TN-FP*FN}{\sqrt{\left(TP+FP\right)\left(TP+FN\right)\left(TN+FP\right)\left(TN+FN\right)}} \end{array}$$

where *N* is the number of all test images, A is accuracy, P is precision, R is recall rate, F1 is a score, *i* is the *i*th type of disease, and *TP*_*i*_, *FP*_*i*_, and *FN*_*i*_ are the numbers of true positives, false positives, and false negatives, respectively, in the *i*th type of disease. MCC is essentially the correlation coefficient between the observed and predicted binary classifications; it returns a value between −1 and +1. The coefficient +1 means perfect prediction, 0 means no better than random prediction, and −1 means complete discrepancy between prediction and observation.

Loss value is another indicator to evaluate the models. Different from the other indicators, loss is an evaluation of the fitting degree of the training set instead of test set. Although it cannot directly represent the performance of the model, the fitting condition of the model can be estimated through the changes in loss during the training process Here, we selected the cross entropy loss function (De Boer et al., 2005).

#### Fine-Tuning of the Models

The models were fine-tuned using the transfer learning method to reduce training time. Transfer learning means applying the knowledge learned from one dataset to another, which has been proven to be effective for plant disease recognition (Kaya et al., 2019; Chen et al., 2020). In transfer learning, models fully trained on the ImageNet dataset were trained again on the rice disease dataset. Since 1,000 classes of ImageNet do not correspond to the number of disease categories identified for rice crop in this study, the last layers of all the models were modified to output six classes. Therefore, before the training for rice diseases, the parameters of the models were set as the pre-trained models except for the last layers. The weights of the last layers were initialized with the method used by He et al. (2015), and biases of the last layers were modified by uniform distribution.

After the pre-training, the models trained using the rice disease dataset were able to extract basic features such as edges and contours of leaves and spots; thus, the models could converge faster. The training policies of the five models were the same, where the batch size was 64, the data loader process number was eight, the max epoch was 200, the optimizer was stochastic gradient decent (SGD) with 0.9 momentum, and the initial learning rate was 0.001. To make the model converge quickly in the early stage and continue to improve in the later stage, a variable learning rate was applied. In the first five epochs, warm up was used, i.e., the learning rate increased linearly from 0 to the initial learning rate, which enabled the model to stabilize rapidly on a large data set. Subsequently, the learning rate decreased to 0 after 30 epochs according to the cosine function, and then returned to the initial learning rate, which decreased repeatedly until the max epoch was reached.

#### Ensemble Learning

**Figure 3:**
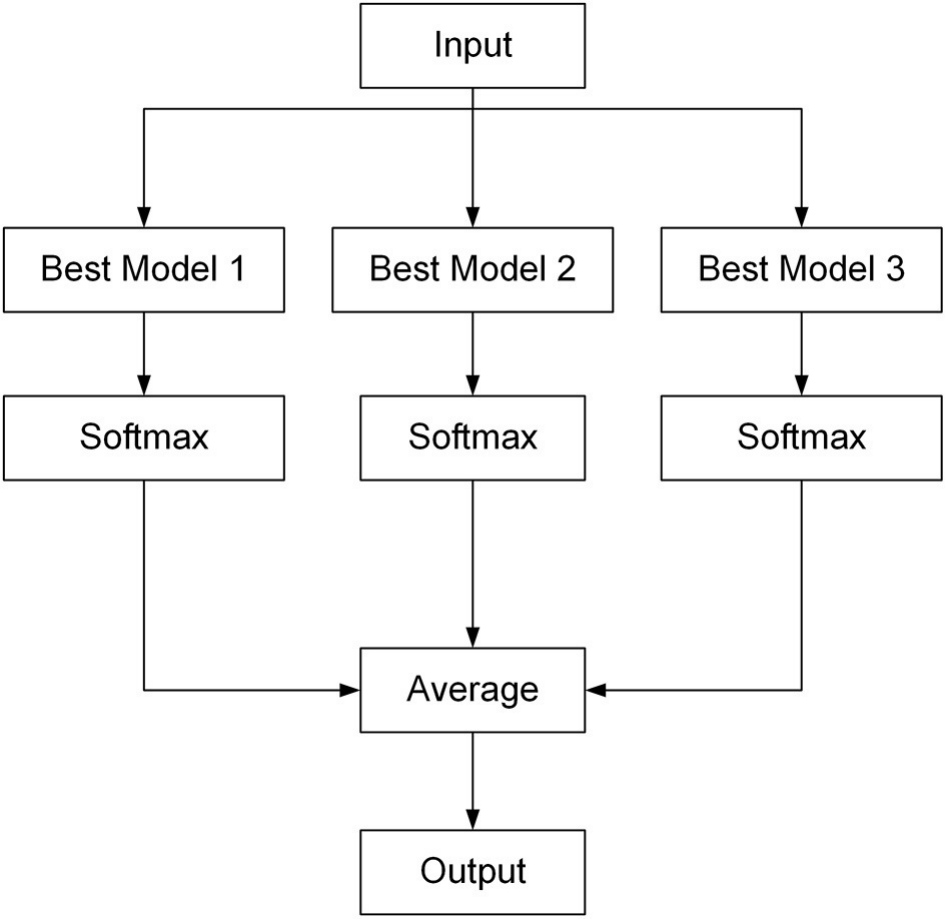
Architecture of the Ensemble Model for rice disease diagnosis.

Ensemble learning combines multiple submodels into a single model so if a submodel fails, the others can correct the errors (Caruana et al., 2004). In this study, ensemble learning was achieved by combining the three best network submodels, which were selected out of the five submodels after comparisons of the performance of the five submodels. The type of the ensemble algorithm implemented here was voting. For the output of each selected network submodel, the Softmax function (Equation 6) was used to normalize first, and then the output scores of all three submodels were averaged to obtain the final scores of all classes, as illustrated in Figure 3. The class that had the highest score was the diagnosed disease for the input image.

(6) $$\begin{array}{r} \sigma {\left(\text{z}\right)}_{i}=\frac{e^{z_{i}}}{\sum_{j=1}^{K}e^{z_{j}}} \end{array}$$

where **z** is a vector of K real numbers, *z*_*i*_ and *z*_*j*_ are the *i*th and *j*th number of **z** respectively, and σ(**z**) is the output vector whose value is between 0 and 1.

### Model Implementation and Application

**Figure 4:**
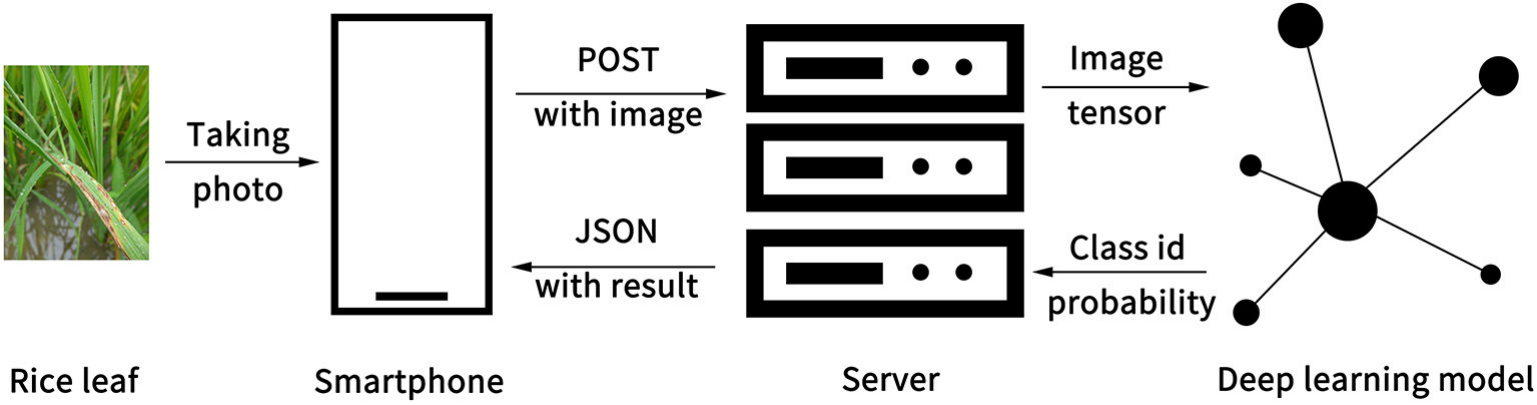
Software architecture of the system.

The Ensemble Model was implemented in an app consisting of software architecture and user interface. The software system had two parts: the client and the server. The client runs on the smartphone, while the server runs on a server computer. As the Ensemble Model was trained and run under PyTorch 1.5.0 with CUDA 9.2 that is based on the Python language (Paszke et al., 2019), the Python language was chosen for the server-side development. Django, a Python-based free and open-source web framework, was used to build a stable web server. The client transmits a rice disease image to the web server. When the server receives a POST request from the client, the server invokes the Ensemble Model to detect the image and returns results to the client in JSON format (Figure 4). The results include status information, disease category, and probability score. After the client receives the JSON data, it parses and displays the data on the screen for the client to view. This structure of front end and back-end separation can help with subsequent functional expansion and support for more platforms in future development.

The user interface for the mobile client was written using Flutter. Flutter is a cross-platform open-source software kit developed by Google, which can be used to develop applications for Android, iOS, Windows, Mac, Linux, and Google Fuchsia. Therefore, the app developed in this study can be used in the Android platform and also in other operating systems after some compilations.

To test the generalization of the Ensemble Model, the app was utilized to recognize rice diseases using a different test set of rice disease images sourced from Google and provided by Shenzhen SenseAgro Technology Co. Ltd. (Shenzhen, Guangdong, China). This set of images includes 50 images for each of the six types of disease, totaling 300 images. With these images, the performance of the Ensemble Model in practical application was evaluated. For the purpose of distinction, this image set was called independent test set, while the images from the original data set was called split test set.

## Results

### Model Training and Testing Results

#### Performance Comparisons of the Five Network Submodels

**Figure 5:**
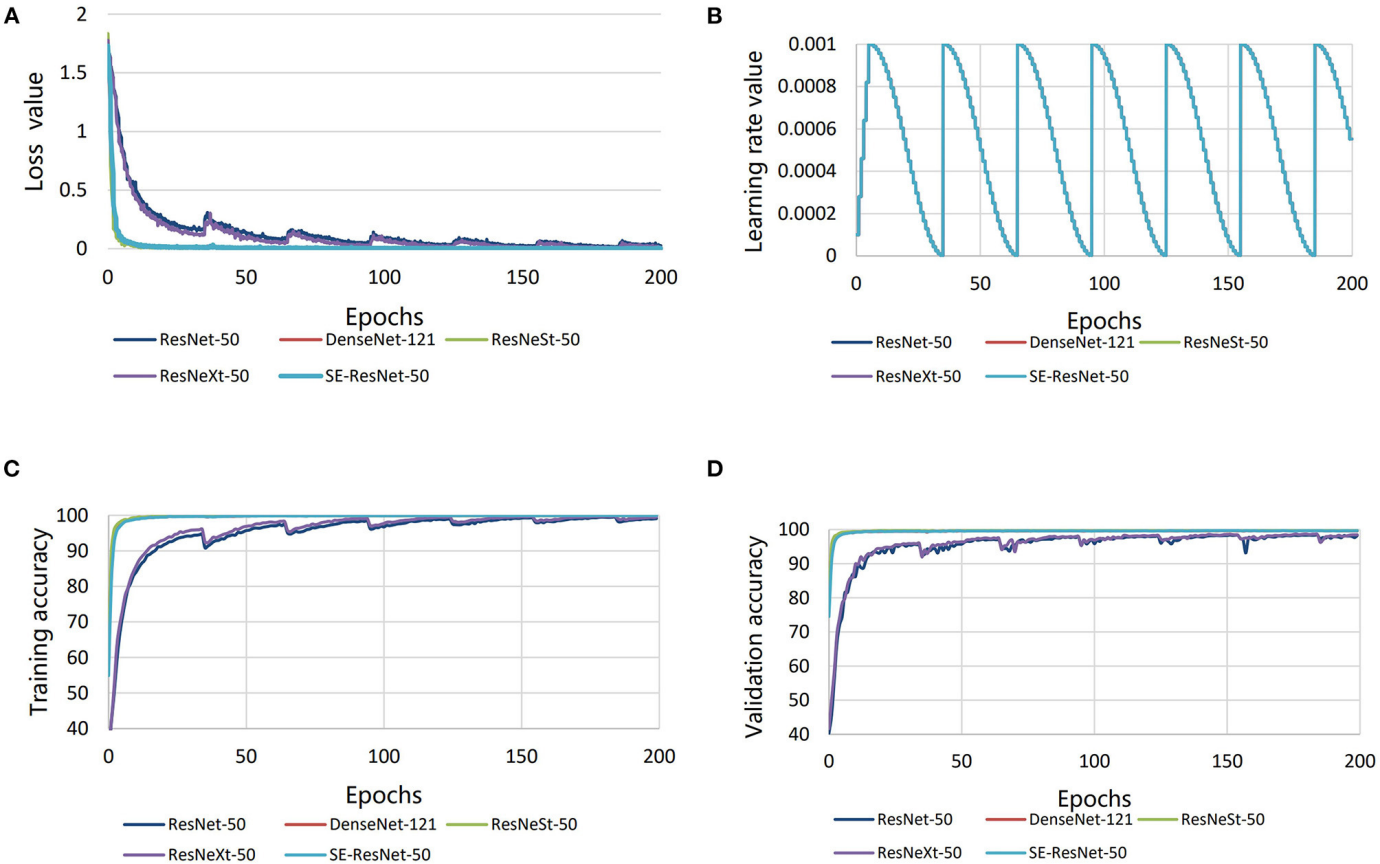
Comparisons in performance of the five different submodels. **(A)** Loss value, **(B)** learning rate, **(C)** validation accuracy, and **(D)** training accuracy.

After fine-tuning and training, the loss value was low for all the five submodels, and the minimum loss values of all the submodels were below 0.002 (Figure 5A). The learning rate was the same for all the submodels, and it was in the range of 0–0.001 (Figure 5B). The disease diagnosis accuracy on the training set of rice disease images was high for all the submodels, meaning all the submodels had fit the training set well, but that SE-ResNet-50, DenseNet-121, and ResNeSt-50 had better accuracies (over 99%) (Figure 5C). When the submodels were applied on the validation set and test set of images, the disease diagnosis accuracy was also high for all the submodels, particularly for the SE-ResNet-50, DenseNet-121, and ResNeSt-50 submodels, which achieved accuracies of over 99% (Figure 5D).

**Figure 6:**
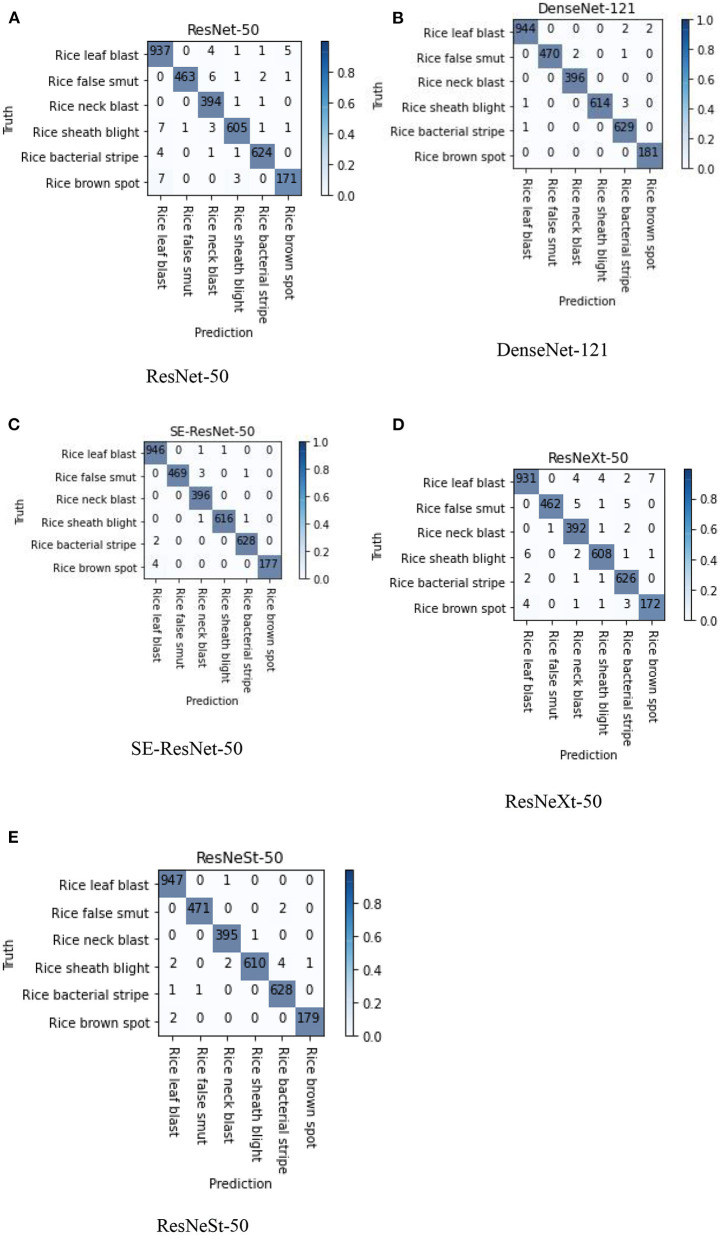
Confusion matrixes of the five different submodels; images used were from the split test set. **(A)** ResNet-50, **(B)** DenseNet-121, **(C)** SE-ResNet-50, **(D)** ResNeXt-50, and **(E)** ResNeSt-50.

Confusion matrix is a specific table that makes it easy to see if the model is mislabeling one class as another. The performance of the five submodels can be visualized using the confusion matrix. Figure 6 shows the confusion matrixes in the split test set of images for the six types of rice diseases. The rows of confusion matrixes are the actual types of disease, while the columns are the predicted type of disease. The diagonal values represent the correct recognition from the model in the categories of true positives (TP) and true negative (TN). The off-diagonal values represent the incorrect recognition in the categories of false positives (FP) and false negative (FN), and smaller values means fewer misrecognitions occurred. The diagonal values were large, and the other values were small, which showed that all the submodels were quite effective in diagnosing all the various types of rice diseases. The depth of the color indicates the proportion of the number at that position to the total of the row, therefore the color on the diagonal represents the recall rate of the disease. According to the confusion matrix, the DenseNet-121, SE-ResNet-50, and ResNeSt-50 submodels overperformed the other two submodels in the confusion of different diseases, especially for the leaf blast, false smut, and sheath blight rice diseases.

To further verify the effect of the confusion matrix results, the MCC of the diseases corresponding to each model were also calculated, as shown in Table 2. According to the MCC values, which are shown in Table 2, the DenseNet-121, SE-ResNet-50, and ResNeSt-50 submodels overperformed the other two submodels in the confusion of different diseases, especially for the leaf blast, false smut, and sheath blight rice diseases.

**Table 2:** MCC values of the five different submodels.

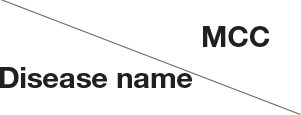	**ResNet-50**	**DenseNet-121**	**SE-ResNet-50**	**ResNeXt-50**	**ResNeSt-50**
Rice leaf blast	0.978	0.995	0.994	0.978	0.995
Rice false blast	0.986	0.996	0.995	0.985	0.996
Rice neck blast	0.977	0.997	0.993	0.976	0.994
Rice sheath blight	0.979	0.996	0.997	0.982	0.990
Rice bacterial stripe	0.989	0.993	0.996	0.983	0.992
Rice brown spot	0.949	0.994	0.988	0.950	0.991

**Figure 7:**
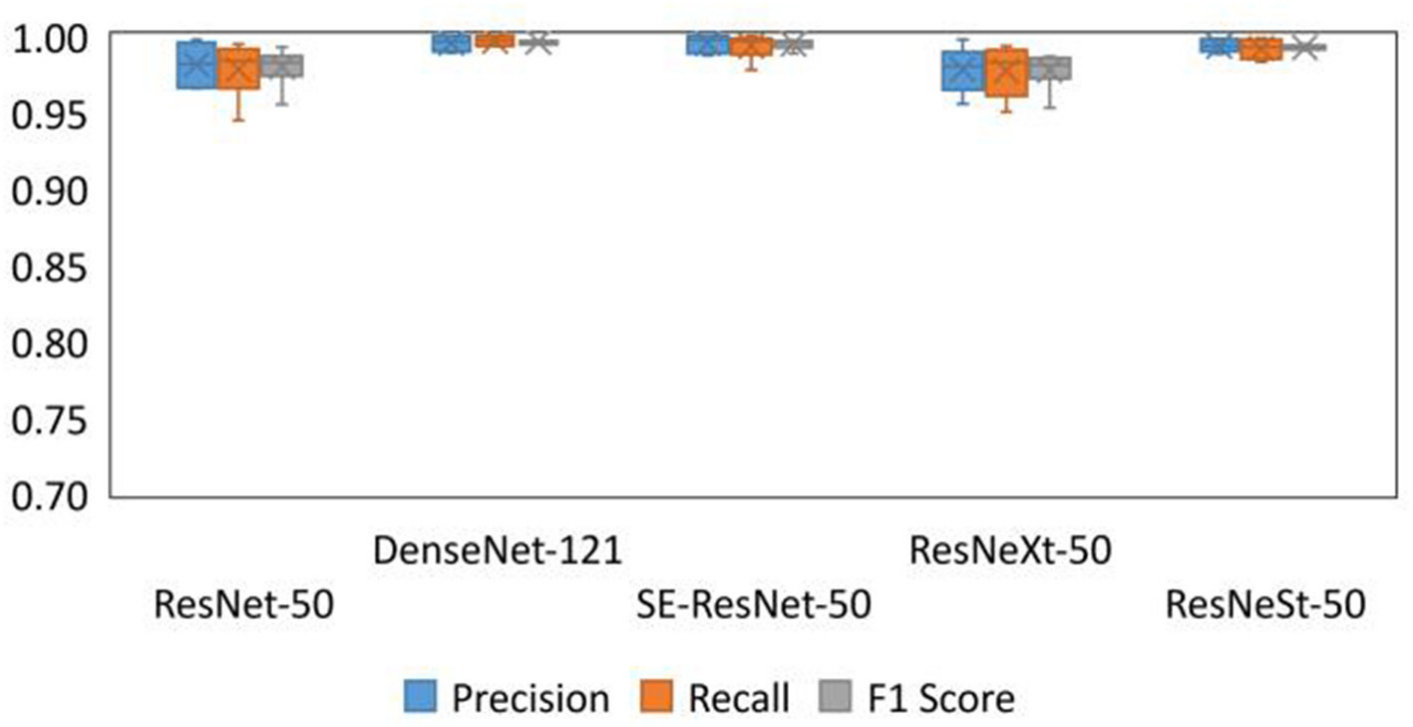
Boxplots of precision, recall, and F1 score of the different submodels.

The precision, recall, and F1 score of each submodel on recognition of each disease were determined using Equations (2–4). Figure 7 below visually compares the boxplots of precision, recall and F1 score values for each of the five models, namely, ResNet-50, DenseNet-121, SE-ResNet-50, ResNeXt-50, and ResNeSt-50. The boxplots suggest that the DenseNet-121 model is significantly better than the other four submodels, whether it is compared with precision, recall, or F1 score. Except for the DenseNet-121 model, SE-ResNet-50 and ResNeSt-50 are better than ResNet-50 and ResNeXt-50 in terms of precision or recall and F1 score. In summary, DenseNet-121, ResNeSt-50, and SE-ResNet-50 had better overall performance among the five submodels tested.

#### Visualization of the Three Best Submodels

**Figure 8:**
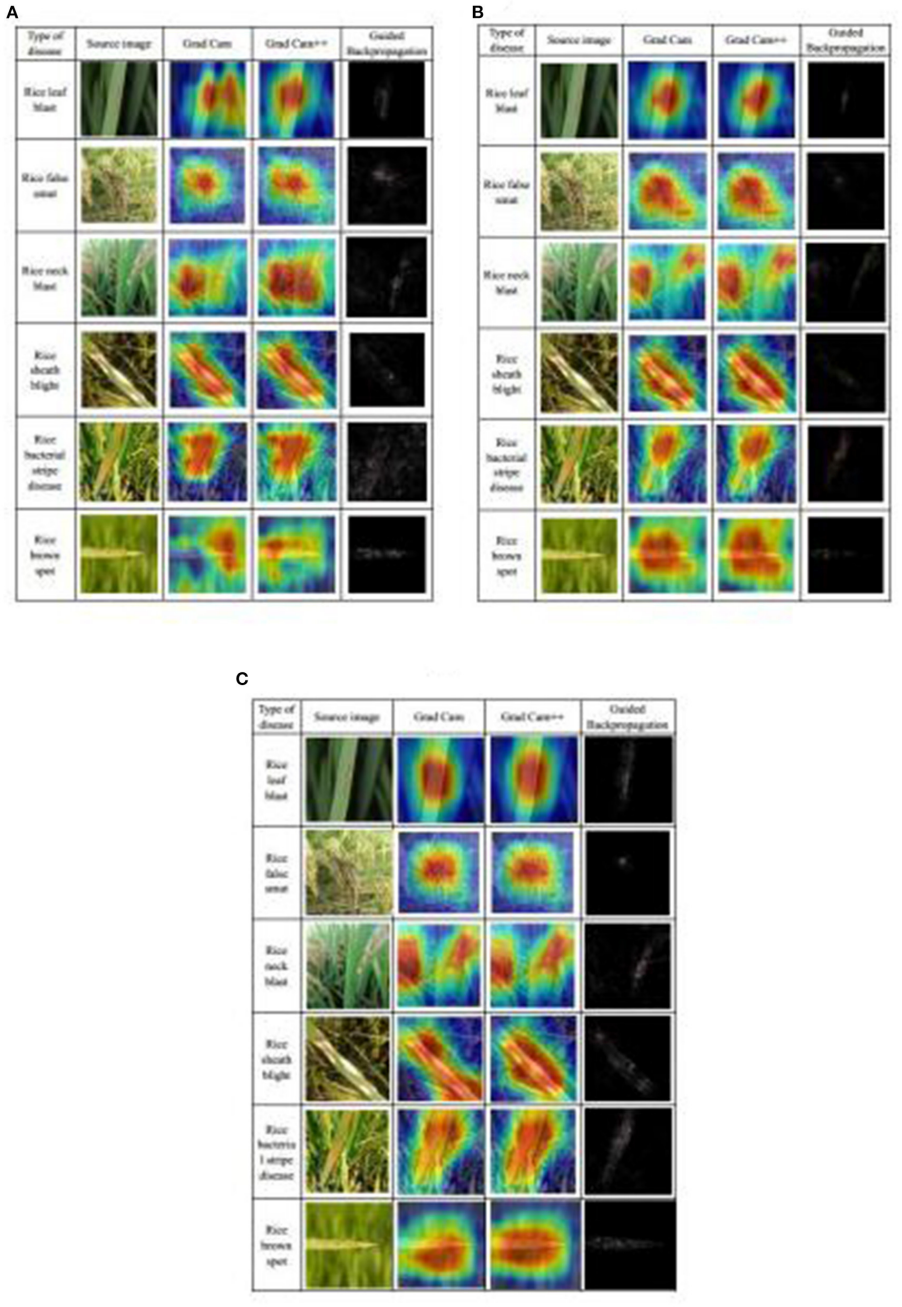
Visualization of rice disease diagnosis results from the three best submodels: **(A)** DenseNet-121, **(B)** ResNeSt-50, and **(C)** SE-ResNet-50.

Based on the discussion above, the three best submodels were DenseNet-121, ResNeSt-50, and SE-ResNet-50. Their performance was further demonstrated by visualization methods: Grad CAM (Selvaraju et al., 2016), Grad CAM++ (Chattopadhyay et al., 2017), and Guided Backpropagation (Springenberg et al., 2015). The CAM is class activation map, which can show the areas most relevant to a particular category and map them to the original image (Zhou et al., 2015). The Grad CAM is calculated by the weighted sum of the feature map and the weight of the corresponding class, which can generate CAM without changing the structure of model. Grad CAM++ is an improved version of Grad CAM, which introduces the weighting of the output gradient for the pixel level at a particular location, and it has better effects than Grad CAM. Guided Backpropagation uses backpropagation to calculate the output-to-input gradient, and it restricts the backpropagation of gradients less than 0 to find the points of the picture that maximizes the activation of a feature. In the results, these points are usually represented as the contours of features. Also, to make the Guided Backpropagation images clearer, high-pass filters using the Sobel operator were taken to post-process the images. The maps of these three visualization methods were generated for each of the three selected submodels on each of the six types of diseases (Figure 8). In the Grad CAM and Grad CAM++maps, the red area represented activation areas, and the model paid more attention to this area in the diagnosing process, whereas the blue area had no positive effect on the result. In the Guided Backpropagation map, the contours, in which the model was interested, were highlighted. It is obvious to find the basis of diagnosis using this map. When comparing the maps among the three submodels, the general shapes and locations of active areas (red areas) in the Grad CAM and Grad CAM++ maps are similar. However, the boundaries of the active areas from DenseNet-121 (Figure 8A) are not as defined as those from the two other submodels (Figures 8B,C). Also, it seemed that the locations of the active areas from SE-ResNet-50 better reflect the disease locations shown in the original images (Figure 8C). In the Guided Backpropagation map, contours of interesting objects from DenseNet-121 (Figure 8A) are not as obvious as those from ResNeSt-50 (Figure 8B), and those from SE-ResNet-50 (Figure 8C) are intermediate in this regard. Overall, all the three selected submodels have a good disease identification ability, as visually observed, and they would complement each other in the Ensemble Model.

#### Performance of the Ensemble Model

**Figure 9:**
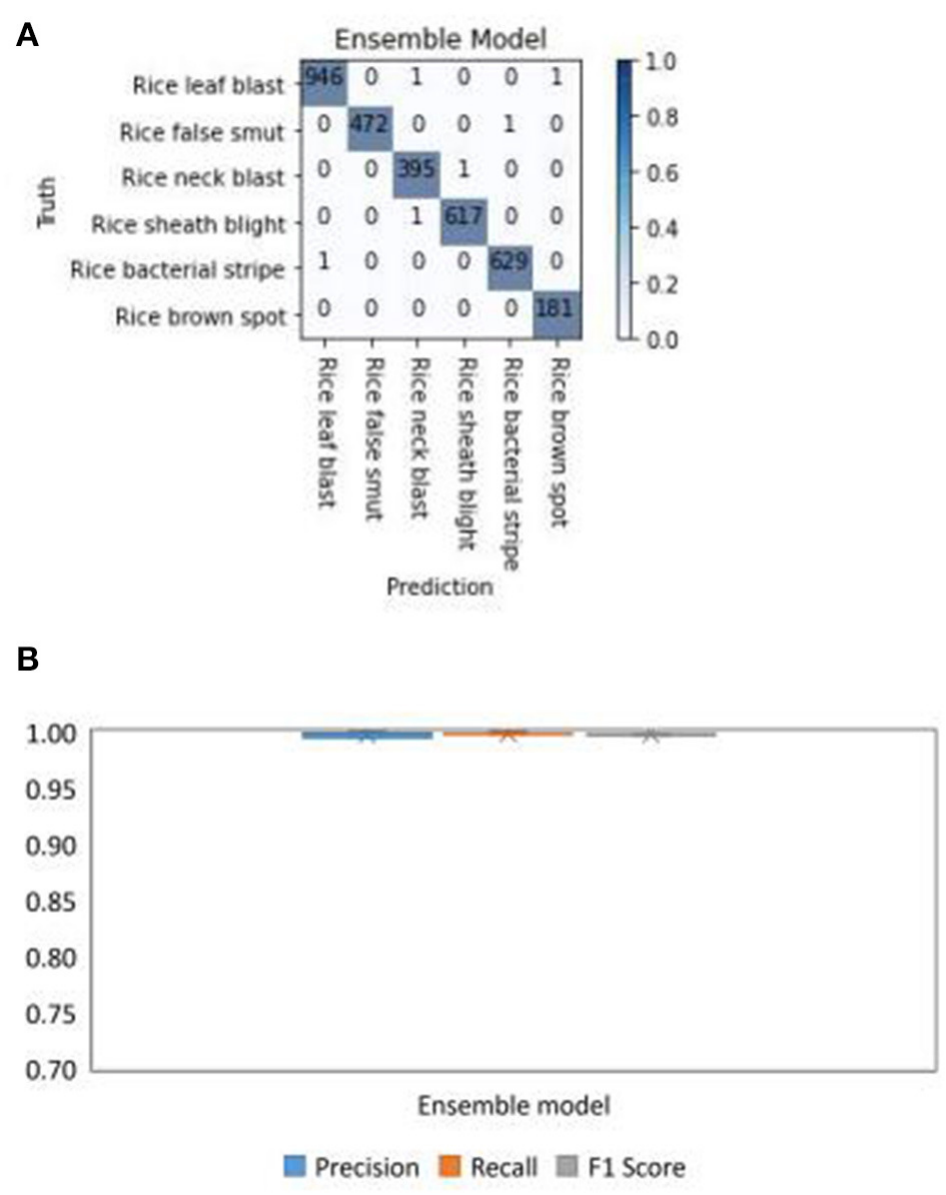
Test results of the Ensemble Model for different types of rice disease with the split test set of images. **(A)** The confusion matrix and **(B)** the boxplots of the precision, recall, and F1 score for the Ensemble model in diagnosing six rice diseases.

To show the performance of the Ensemble Model, which is a combination of DenseNet-121, ResNeSt-50, and SE-ResNet-50, the confusion matrix was calculated. The diagonals of the confusion matrix indicated high values of TP (Figure 9A), meaning the Ensemble Model had an accuracy of over 99%. The boxplots of the performance indicators of the Ensemble Model: precision, recall, and F1 score, are shown in Figure 9B. The boxplots show that the Ensemble Model did not have outliers in precision, recall, and F1, indicating that the performance of the model in identifying diseases is very stable. These results demonstrate that the Ensemble Model had a good performance in recognizing all the six types of rice diseases.

### Application of the Ensemble Model

**Figure 10:**
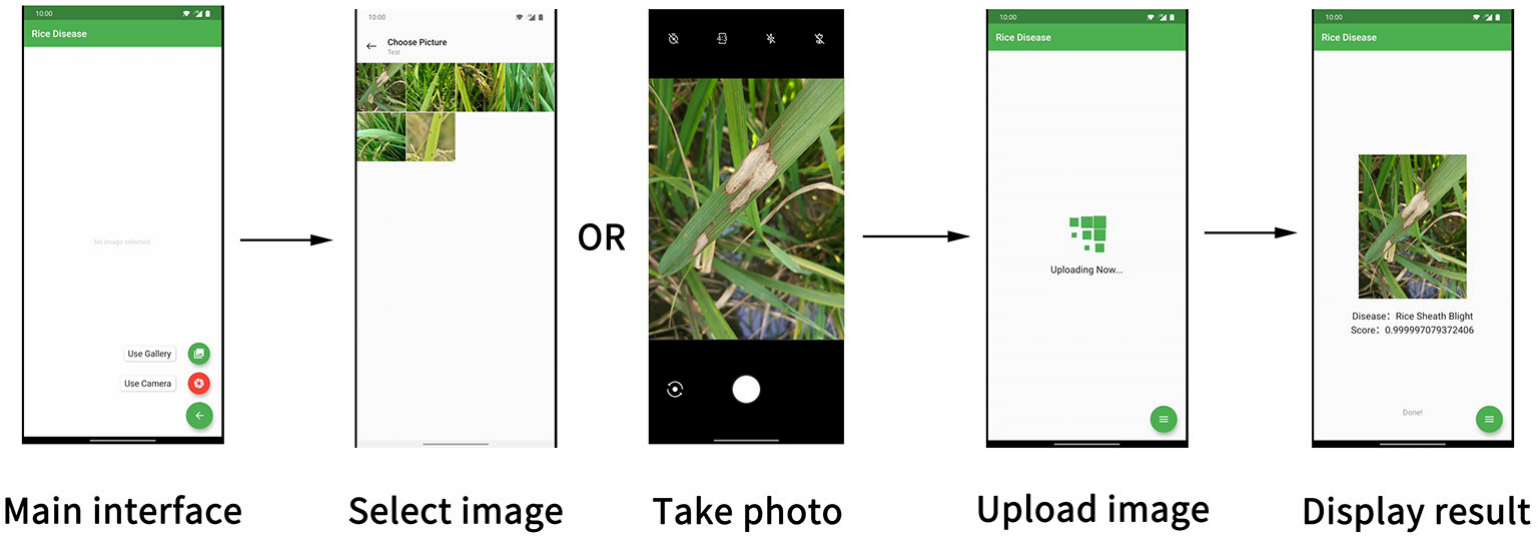
Components of the user interface in the rice disease diagnosis app.

In the rice disease diagnosis app, the user interface is composed of several parts, as shown in Figure 10. The main interface was for taking photos or uploading existing pictures. The photo interface was used for taking disease images and uploading them. The picture-selecting interface was used to select the existing disease pictures in the mobile phone for uploading. Considering the time required for network uploading, a wait interface was provided to improve user experience. After the client received the data returned by the server, the result interface displayed the results of the recognition of the disease image by the model.

**Figure 11:**
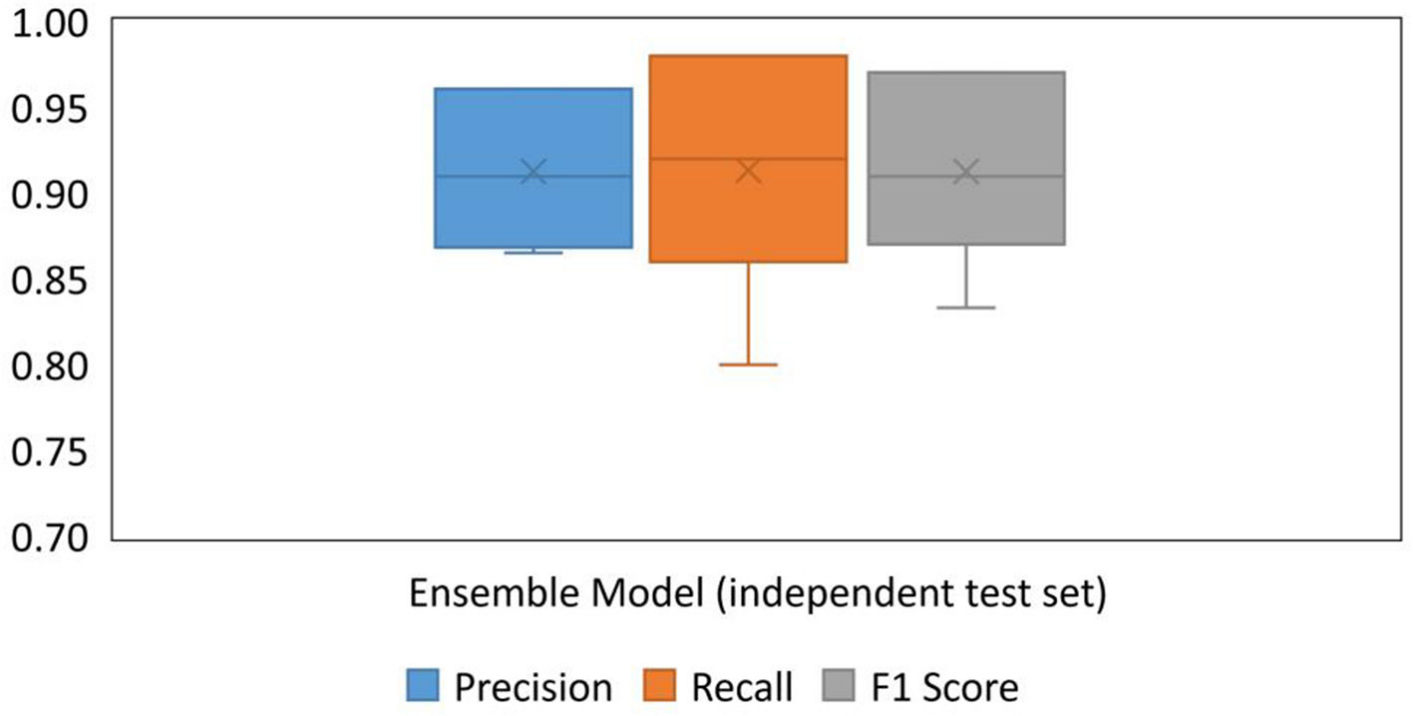
Boxplots of precision, recall, and F1 score for the Ensemble Model, tested with the independent test set of images.

To test the performance of the app in a practical application, a test set of images from different sources (Google images and SenseAgro) was used to verify the generalization of the Ensemble Model and the performance of the app. The boxplots of precision, recall, and F1 scores for the Ensemble Model are shown in Figure 11. The boxplots illustrate that the Ensemble Model had a small degree of dispersion in precision, recall, and F1 score, indicating that the performance of the model in identifying diseases is relatively stable. The F1 score varied from 0.83 to 0.97 when the Ensemble Model was used to diagnose different types of disease. As for the overall performance, the results showed that the accuracy for all the diseases was 91%. As the F1 scores are over 0.8 and the accuracy is over 90% for all the diseases, the rice disease diagnosis app is considered to be good.

## Discussion

Rice leaf blast, rice false smut, rice neck blast, rice sheath blight, rice bacterial stripe, and rice brown spot are common diseases during the growth of rice. The identification of these diseases is of practical importance and can provide ideas for the identification of other rice diseases in the future. In this study, the dataset was split into a training set, a validation set, and a test set using a ratio of 7:2:1. From the training results, the ratio made full use of the data obtained from the collection and enabled the model to learn the important features of each disease. Considering that the test set obtained from splitting this dataset has a large similarity with the training set, various disease images from different sources were collected to form an independent test set. The test results of the independent test set demonstrate that the network designed in this study is generalizable and can be applied in practice. Therefore, the division of the data set and the selection of the test set are appropriate for this study.

### Comparison of the Submodels

The convergence speeds of DenseNet-121, ResNeSt-50, and SE-ResNet-50 were high (Figure 5), and they reached a stable level when about 30 epochs were iterated, while ResNet-50 and ResNeXt-50 were relatively stable after 100 epochs. Throughout all the training processes, DenseNet-121, ResNeSt-50, and SE-ResNet-50 were more accurate than ResNet-50 and ResNeXt-50. The accuracy curves and the loss curves of the three submodels were also smoother. This indicates that DenseNet-121, ResNeSt-50, and SE-ResNet-50 have faster convergence speeds, higher accuracy rates, and more stable convergence states.

The confusion matrixes show that most diagnosis results were correct, and that some diseases were more easily misrecognized than the others (Figure 6). There was a confusion between rice leaf blast and brown spot diseases in some of the submodels, because the early characteristics of rice leaf blast and rice brown spot were very similar. Both diseases consist of small brown spots, which are difficult to distinguish by naked eyes. Rice false smut and rice neck blast are also easily confused because they both appear at the ear of rice, which could sometimes lead to misjudgment by the submodels.

Figure 7 provides a more intuitive view of the performance of the different submodels on different diseases. DenseNet-121, ResNeSt-50, and SE-ResNet-50 perform better than the other two submodels; the gap is most pronounced in rice brown spot. Each of the three submodels have internal advantages for different diseases. DenseNet-121 performed better with rice neck blast and rice brown spot; SE-ResNet-50 performed better with rice bacterial stripe; and ResNeSt-50 was more balanced with different diseases. Therefore, considering the better performance of DenseNet-121, ResNeSt-50, and SE-ResNet-50, these three submodels were selected as the submodels of the Ensemble Model.

### Visualization Analysis of the Models

The learning conditions of different networks to different diseases can be found (Figure 8). For rice leaf blast disease, characterized by large spindle-shaped lesions with grayish centers and brown edges, all three submodels are more sensitive to the whole spot area, so all of them could accurately learn the characteristics of this disease. In detail, the areas on Grad CAM and Grad CAM++ of ResNeSt-50 were the most precise, and in the Guided Backpropagation maps, the spots were the most obvious. Therefore, the feature extraction of ResNeSt-50 for rice blast was the best.

For rice false smut disease, the pathogen is fungal that infects rice flowers and turns them into rice false smut balls, which are the only visible feature of rice false smut. The heatmap of the three submodels is very close, the part that includes the rice false smut ball is focused, while the surrounding normal rice is ignored, which means that the learned characteristics of rice false smut are the same.

For rice neck blast disease, node and neck lesions often occur at the same time and have a similar characteristic, a blackish to a grayish brown color. DenseNet-121 and SE-ResNet-50 mainly focus on the neck and node of rice, while ResNeSt-50 mainly focus on the node of rice, which means that the feature extraction ability of ResNeSt-50' in rice neck blast is poor compared with the other two submodels, as the latter submodel did not fully learn all the characteristics in the node and neck.

For rice sheath blight disease, lesions on the leaves are usually irregular in shape, and after a period of infection, the center is usually grayish-white, and the edges are usually brown. The Grad Cam heatmaps of the three submodels are also similar, and all the lesions are of concern.

For rice bacterial stripe disease, on young lesions, the bacteria ooze dew and dry the plant out, leaving yellow beads that eventually develop orange-yellow stripes on the leaves. DenseNet-121 and SE-ResNet-50 focus on most of the spots, while ResNeSt-50 focuses only on the upper spots, which means ResNeSt-50 is weaker than the other two submodels in feature extraction of rice bacterial stripe disease.

For rice brown spot disease, the spots are initially small round, dark brown to purplish brown, and fully developed spots are round to elliptic with light brown to gray centers and reddish-brown edges. DenseNet-121 performs poorly in feature learning and is only sensitive to some features, while the other two submodels contain most of the disease spots.

It should be noted that these heatmaps can only indicate which features the model paid more attention to, indicating that the model learned the features of the spots rather than other unrelated features. However, this is not exactly consistent with the final classification score of the model, because different types of diseases interact with each other. It is not enough to learn the characteristics of a disease. Learning the characteristics of the differences between various diseases also affects the final classification performance. Therefore, although the heatmaps of some models are not perfect for some diseases, they can still be well-classified.

### Performance of the Ensemble Model

The results of the Ensemble Model tested with the split test set of images (Figure 9) showed that by combining the scores of the different models, the confusion between different diseases was greatly reduced. This explains that the Ensemble Model combines the advantages of each model to solve the problem of a single model misjudging some diseases. Meanwhile, the precision, recall, and F1 scores of the Ensemble Model were also more stable than those of the single model.

The F1 scores of the Ensemble Model for each disease were tested using the independent test set of images, and the overall accuracy of the Ensemble Model in the independent test set was 91% (Figure 11). Compared with the results of the previous test in the split test set, it can be found that although there was a reduction in accuracy, it was still high. The best recognition effect was on the rice sheath blight and rice bacterial stripe diseases; their indicator scores were close to one, which was close to the results from the test using the split test set of images. This means that the Ensemble Model has the best generalization for these two diseases. The indicators of rice leaf blast, rice false smut, and rice neck blast were all around 0.9, which was mainly caused by the confusion between diseases, and the samples from different sources also had some influence. The F1 score of brown spot disease was close to 0.8. On one hand, the training samples of rice brown spot were least in all the diseases, although data enhancement was performed. On the other hand, rice leaf blast and rice brown spot have similar characteristics, which may cause confusion easily. In general, the performance of the Ensemble Model in the independent test set was satisfactory, which indicated that the rice disease diagnosis app is reliable to be applied in the field.

Since the dataset used for training and testing in this study is different from that in previous studies and the diseases targeted by the study are different, a direct comparison cannot be made. However, the Ensemble Model designed in this study performed better on the split test set than the previous study on the corresponding dataset (Lu et al., 2017b; Rahman et al., 2020), which indicates that the Ensemble Model designed in this study is effective. The results on the independent test set also demonstrate the good generalization of the Ensemble Model. Therefore, as compared with previous applications, the proposed smartphone app can provide higher accuracy, which is the most important performance indicator of the application. To facilitate the implementation of the app, easy operation and simplicity are the key features for farmers to quickly adopt the app. Finally, the cost is a barrier to commercialization of any technology. The low cost of the app will attract many users.

## Conclusion

In this study, a dataset containing 33,026 images of six types of rice diseases was established. Based on these images, five submodels, ResNet-50, ResNeXt-50, DenseNet-121, ResNeSt-50, and SE-ResNet-50 were trained and tested, achieving over 98% accuracy and over 0.95 F1 score. Among them, DenseNet-121, SE-ResNet-50, and ResNeSt-50 performed well. Visual analysis confirmed the good learning status of the submodels on the characteristics of rice diseases. Subsequently, the Ensemble Model, an integration of these three submodels, produced accurate judgment of confusable diseases, according to the confusion matrixes analysis. As a result, the F1 scores reached more than 0.99 for each of the six types of disease. Being tested by independently sourced images, the Ensemble Model achieved 91% accuracy, indicating that it has enough generalization ability to be implemented in a rice disease diagnosis app for field applications. With a software system that included both servers and clients, the smartphone app provided high accuracy, easy operation, simplicity, and low-cost means for the recognition of rice diseases. The limitation was that the Ensemble Model has many parameters, which may affect the speed of identification. Future studies will be carried out on network pruning to reduce the number of parameters.

## Data Availability Statement

The raw data supporting the conclusions of this article will be made available by the authors, without undue reservation.

## Author Contributions

RD conceptualized the experiment, selected the algorithms, collected and analyzed the data, and wrote the manuscript. MT trained the algorithms, collected and analyzed data, and wrote the manuscript. HX analyzed the data. CL and KL collected the data. XY and LQ supervised the project. All the authors discussed and revised the manuscript.

## Conflict of Interest

The authors declare that the research was conducted in the absence of any commercial or financial relationships that could be construed as a potential conflict of interest.

## Publisher's Note

All claims expressed in this article are solely those of the authors and do not necessarily represent those of their affiliated organizations, or those of the publisher, the editors and the reviewers. Any product that may be evaluated in this article, or claim that may be made by its manufacturer, is not guaranteed or endorsed by the publisher.

## Supplementary Material

Supplementary Figure 1 Sample images illustrating disease levels.
