# Supplementary figures and images for: Automatic Diagnosis of Rice Diseases Using Deep Learning
Source: Front Plant Sci. 2021 Aug 19;12:701038. doi: 10.3389/fpls.2021.701038 (PMC8416767; doi:10.3389/fpls.2021.701038)

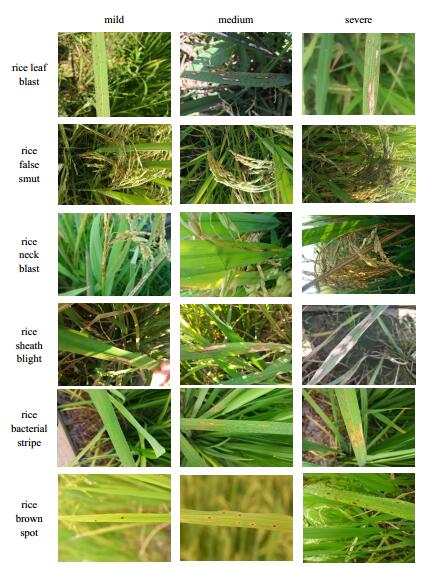

Supplement: Supplementary Figure 1 — Sample images illustrating disease levels. [file Image_1.JPEG]
